# Evaluation of serum irisin level and severity of erectile dysfunction in diabetic males: a cross sectional prospective study

**DOI:** 10.1186/s13098-024-01452-3

**Published:** 2024-09-19

**Authors:** Ahmed Ragab, Ahmed Reda Sayed, Sameh Fayek GamalEl Din, Ashraf Zeidan, Faten Fathi Ewis, Mostafa Ahmed Hamed

**Affiliations:** 1 Department of Andrology, Sexology and STDs, Faculty of Medicine, Beni-Suef University, Beni-Suef, Egypt; 2 Department of Medical Biochemistry and Molecular Biology, Faculty of Medicine, Beni-Suef University, Beni-Suef, Egypt; 3 Department of Andrology, Sexology and STDs, KasrAlainy Faculty of Medicine, Cairo University, Al-Saray Street, El Manial, Cairo, 11956 Egypt; 4 Beni-Suef Mental Health Hospital, Beni-Suef, Egypt

**Keywords:** Type 2 diabetes mellitus, Erectile dysfunction, Serum irisin

## Abstract

**Background:**

Irisin is an exercise-induced myokine that alleviates endothelial dysfunction and reduces insulin resistance in type 2 diabetes mellitus (T2DM). The current study aimed to assess the serum level of irisin in T2DM men with erectile dysfunction (ED) compared to T2DM patients with normal erectile function and healthy controls, as well as investigate the association between serum irisin level and the severity of ED in T2DM patients.

**Patients and methods:**

A cross-sectional study was conducted on 90 males, divided into three groups: 32 T2DM patients with ED, 24 T2DM patients without ED, and 34 healthy controls. Socio-demographic characteristics and scores of the validated Arabic version of the international Index of Erectile Function-5 (ArIIEF-5), Generalized Anxiety Disorder-7 (GAD-7) and Patient Health Questionnaire-9 (PHQ-9) were obtained. Furthermore, routine laboratory tests employed for diabetes monitoring and serum levels of total testosterone and irisin were assessed within these groups.

**Results:**

T2DM men with ED had significantly lower serum levels of irisin and testosterone, as well as a lower ArIIEF-5 score, but their GAD-7 and PHQ-9 scores were significantly higher than those without ED or controls (p < 0.001). Among T2DM men, serum irisin levels positively associated with ArIIEF-5 scores and serum testosterone (r = 0.413, p = 0.002; r = 0.936, p < 0.001, respectively) but negatively associated with glycosylated hemoglobin levels (r = -0.377, p = 0.004). Multivariate regression analysis to predict ED in T2DM patients found that GAD-7 score was the only most significant predictor for ED (ꞵ = − 1.176, standard error = 0.062, p < 0.001).

**Conclusion:**

The current study had demonstrated that irisin positively correlated with the ArIIEF-5 and serum testosterone but negatively correlated with HbA1c in T2DM men. Nevertheless, further validation of these findings is necessary through cohort studies.

**Supplementary Information:**

The online version contains supplementary material available at 10.1186/s13098-024-01452-3.

## Introduction

Erectile dysfunction (ED) is defined as a persistent inability to attain and maintain an erection sufficient to have a satisfactory sexual performance [1]. ED is a common complication of diabetes [2]. ED is associated with a reduced quality of life and unfortunately occurs at an earlier age in diabetic patients compared to the general population [2]. In the European Male Ageing Study, the prevalence of moderate or severe ED was 19%, 38%, and 64% among participants who were 50–59, 60–69, and above 70 years of age, respectively [3]. Mechanisms responsible for ED due to endothelial dysfunction associated with DM and metabolic syndrome included low-grade systemic inflammation, fibrosis, and smooth muscle dysfunction. Despite this, mechanisms related to the occurrence of ED in DM and metabolic syndrome had not been fully elucidated [4]. Biological markers could help to explain these mechanisms and could be used as useful indicators for ED [5]. Irisin was a recently discovered myokine that was thought to be associated with metabolic diseases [6]. While at first it was considered to be a hormone that could only be synthesized in skeletal muscle, it was later found to be synthesized in many tissues in the body [6]. A previous in vivo study had shown that an increase in serum irisin levels could cause browning of white adipose tissues and decreased insulin resistance [7]. Moreover, the use of exogenous irisin was thought to alleviate endothelial dysfunction in type 2 diabetes (T2DM) by reducing oxidative and nitrative stresses [8].

The current study aimed to assess serum level of irisin in T2DM men with ED compared to T2DM patients with normal erectile function and healthy controls, as well as investigated the association between serum irisin level and the severity of ED in T2DM males.

## Patients and methods

A cross-sectional study was conducted from July 2022 and February 2023 at the andrology department of Beni-Suef University Hospital in Beni-Suef governorate. The study involved 90 male individuals who were split into 3 groups including 32 ED patients with T2DM (Group I), 24 T2DM patients without ED, and 34 free controls (Group III). The institutional ethical committee in the faculty of medicine at Beni-Seuf University approved the work (Approval Number: FMBSUREC/07062022/Ewis). Additionally, informed consent was obtained from the participants conforming to the guidelines stated in the Helsinki Declaration of 2013 [9].

### Sample size determination

G power was used to determine sample size, and a minimum of 70 patients and 18 controls made up the overall sample size. A priori analysis utilized various inputs to calculate the required sample size, including a two-tailed test, an effect size of 0.67, a α error probability of 0.05, a power of 0.70, and an allocation ratio of 4/1. The outcome was determined by the non-centrality parameter, critical t-value, and degrees of freedom. The final result indicated that a total sample size of 88 was needed, with 18 participants in the control group, 70 participants in the case group, and a real power of 0.71.

### Inclusion criteria for patients

All participants were in a steady relationship with their female partners during the past 6 months. Patients were diagnosed as having T2DM according to the European Association for the Study of Diabetes (EASD) criteria [10], aged 25–65, and referred for follow-up. Patients scoring 22 or more on the Arabic version of the International Index of Erectile Function-5 (IIEF-5) [11] were assigned to T2DM patients with normal erectile function, while those scoring less than or equal to 21 were assigned to T2DM patients with ED.

### Inclusion criteria for the controls

Controls encompassed healthy volunteers matched for age, sex, and body mass index (BMI).

### Exclusion criteria for the patients

The study excluded individuals who had a BMI below 19 kg/m^2^ or above 30 kg/m^2^, history indicating psychogenic ED, comorbidities linked to ED [such as coronary artery disease, neurological diseases, major mental illness, thyroid dysfunction, hypogonadism, active liver disease, end-stage kidney disease, uncontrolled hypertension or hyperlipidemia]. Also, medications that impacted erectile function, cases of Peyronie's disease, a history of pelvic trauma or radical surgery, ongoing infections, or cases engaged in intense physical activity within the last week of sampling were excluded.

The study participants were subjected to the following:

All participants provided their medical and sexual histories. General and local examinations were carried out. During a structured interview, each participant was instructed to independently fill out the questionnaire provided. The participants were evaluated using the validated Arabic version of the IIEF-5 (ArIIEF-5) to determine their erectile function and ED severity [11]. Anxiety symptoms were assessed using the Arabic version of the generalized anxiety disorder-7 (GAD-7) scale [12]. Furthermore, depressive symptoms were assessed using the Arabic version of the Patient Health Questionnaire-9 (PHQ-9) [13]. Five cc venous blood sample was collected after 12 h overnight fasting (between 8 and 9 A.M before any significant physical activity). The samples were processed to isolate the serum, which was then stored at – 20 °C. Standard diabetes monitoring tests including fasting plasma glucose, glycosylated hemoglobin, serum lipid profile, and hepatic and renal function panels were conducted. Furthermore, the serum samples were analyzed for total testosterone and serum irisin levels. Serum testosterone levels were measured using acommercial ELISA kit (MyBiosource, Inc., San Diego, CA,USA) according to the manufacturer’s instructions. The assay kit exhibited low coefficients of variation for both intra- and inter-assay measurements, with values below 15%. The detection range for testosterone was 0.2–80 ng/mL, and the assay demonstrated a sensitivity of < 0.1 ng/ml. For the measurement of serum irisin levels, a human irisin sandwich ELISA kit from ELK Biotechnology Co. (Denver, Colorado, USA) was employed. This kit demonstrated excellent precision with low coefficients of variation for both intra- and inter-assay measurements, with values below 8% and 10%, respectively.

The detection range for irisin was 15.7–1000 pg/mL, and the assay exhibited a sensitivity of 6.9 pg/mL.

### Statistical analysis

Data were coded and entered using the statistical package for the Social Sciences (SPSS) version 28 (IBM Corp., Armonk, NY, USA). Data was summarized using mean and standard deviation for quantitative variables and frequencies and percentages for categorical variables. Normality was double-checked using the Shapiro–Wilk test and normality plots in each of the three groups. Comparisons between groups were done using analysis of variance (ANOVA) with multiple comparisons. post hoc test in normally distributed quantitative variables, while the non-parametric Kruskal–Wallis test and Mann–Whitney test were used for non-normally distributed quantitative variables. χ^2^ test was performed for comparing categorical data. Exact test was used instead when the expected frequency is less than 5. Correlations between quantitative variables were done using the Spearman’s correlation coefficient. The receiver operating characteristic (ROC) curve was constructed with area under curve analysis performed to detect the best cutoff value of significant parameters for the detection of ED.

## Results

As illustrated in Table 1, there were no significant differences in age and BMI as well as the duration of DM and the irisin/testosterone ratio between the study groups. On the other hand, significant differences were observed among all groups regarding glycosylated hemoglobin (HbA1c), serum triglycerides (TGs), serum total cholesterol as well as the levels of serum irisin and total testosterone (Table 1).

**Table 1:** Sociodemographic, clinical and laboratorycharacteristics of the participants

	Group I [DM with ED] n = 32		Group II [DM without ED] n = 24		Group III [Controls] n = 34		P value
	Mean	SD	Mean	SD	Mean	SD	
Age (years)	49.75	7.81	48.13	4.49	46.94	6.31	0.218
DM duration (years)	6.91	3.57	6.25	3.19	-	-	0.571
BMI (kg/m^2^)	24.59	2.69	23.42	2.72	23.38	2.07	0.097
Hb A1c (%)	7.65	0.92	6.22	0.57	4.88	0.22	< 0.001^d^
Serum triglycerides (mg/dl)	149.34	8.57	140.04	10.39	133.29	16.54	< 0.001^c^
Serum total cholesterol (mg/dl)	175.47	24.79	163.42	17.87	160.71	23.26	0.024^a^
Serum irisin (pg/mL)	3.21	2.80	6.03	2.94	8.18	3.05	< 0.001^c^
Serum testosterone (ng/ml)	2.30	1.41	4.40	1.92	5.52	1.45	< 0.001^c^
Irisin/Testosterone ratio	1.26	0.37	1.38	0.38	1.65	1.16	0.125
GAD-7	12.34	2.35	4.17	1.17	2.94	0.89	< 0.001^d^
PHQ-9	6.84	3.47	3.88	1.54	2.85	0.99	< 0.001^c^
ArIIEF-5 score	12.00	3.43	22.17	0.38	22.85	0.78	< 0.001^c^

^a^ Group I differs significantly from group III; ^b^ Group I differs significantly from group II; ^c^ Group I differs significantly from both group II and group III; ^d^ Group I differs significantly from both group II and group III, while group II also shows significant differences from group III

*ArIIEF-5* Arabic version of the international index of erectile function-5, *BMI* body mass index, *DM* diabetes mellitus, *GAD-7* generalized anxiety disorder-7, *HbA1c* glycosylated hemoglobin, *PHQ-9* patient health questionnaire-9

**Fig. 1:**
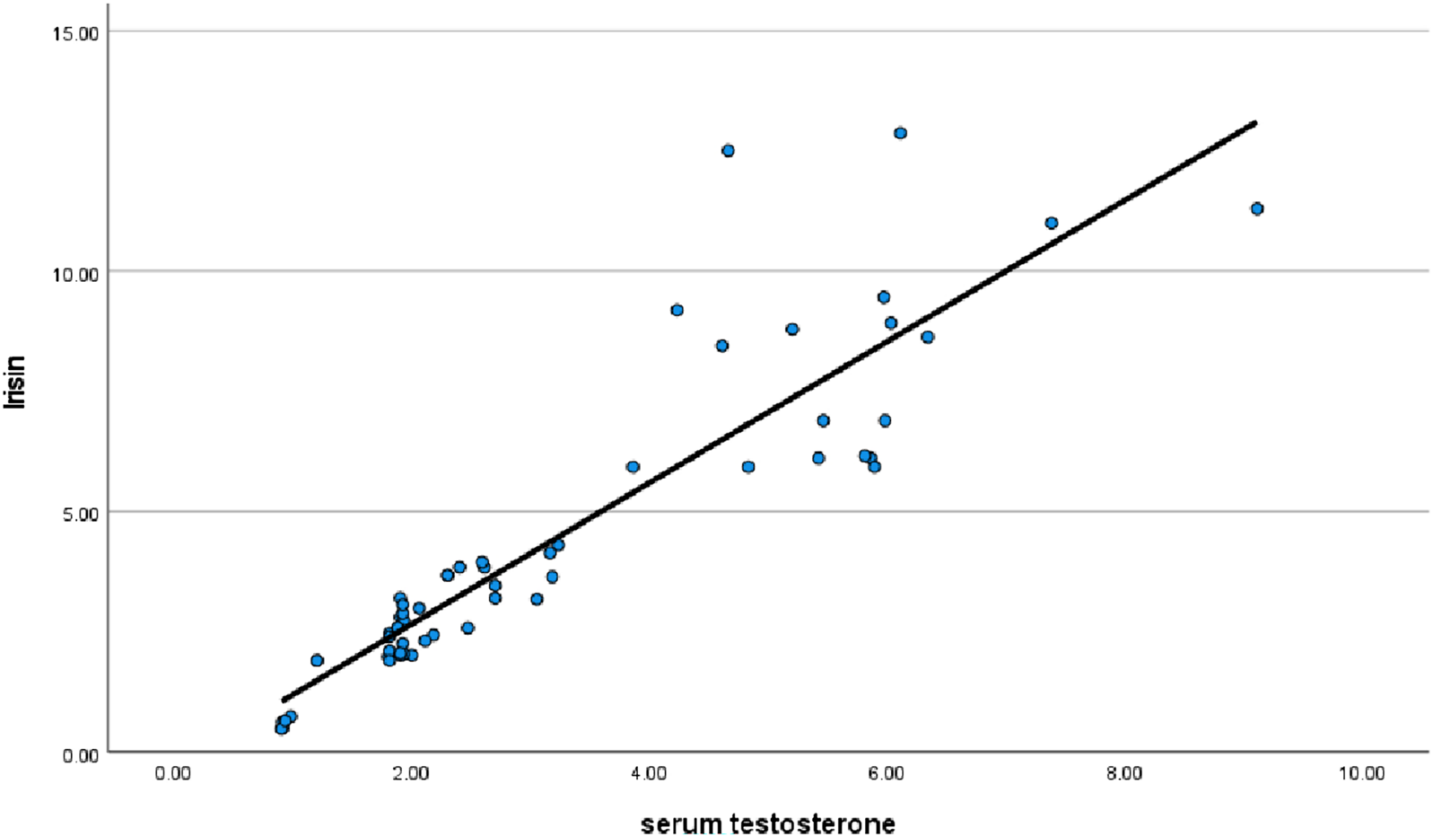
shows correlation between testosterone and irsin in T2DM men

**Fig. 2:**
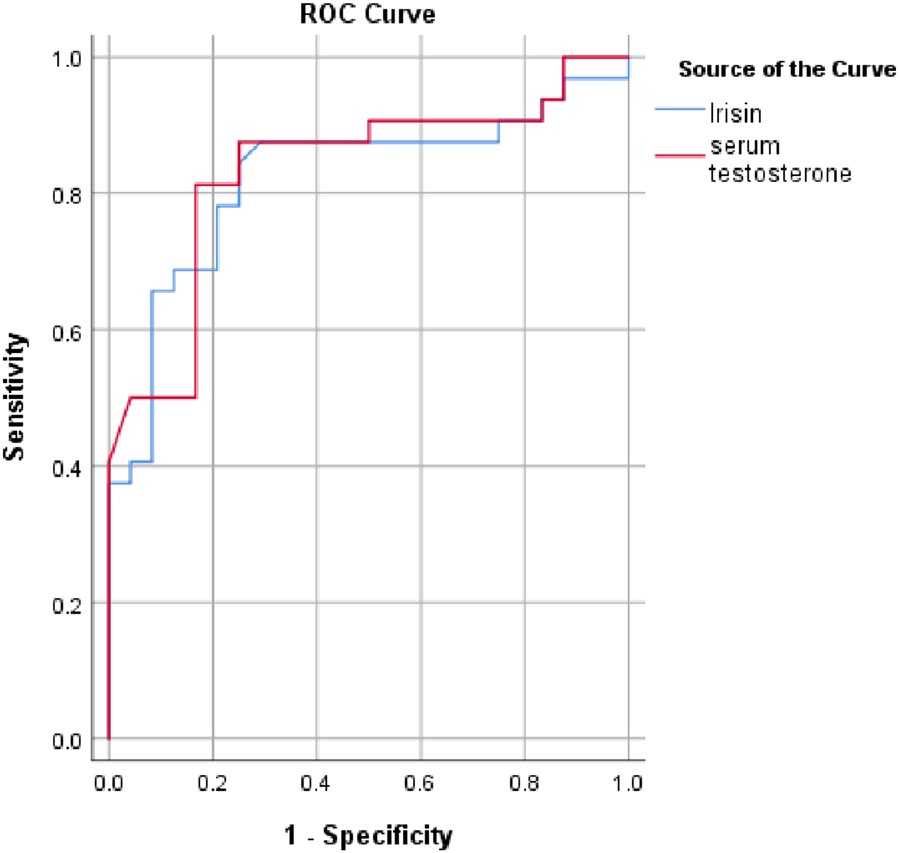
ROC curve for detection of erectile dysfunction in in T2DM patients using irisin and serum testosterone

Regarding GAD-7 and PHQ-9 scores, T2DM patients with ED had significantly higher scores compared to the other groups (Table 1). In contrast, T2DM patients with ED had significantly lower scores of the ArIIEF-5 compared to the other groups (Table 1). The current study revealed significant positive correlations between serum irisin level, ArIIEF-5, DM duration and serum testosterone (r = 0.413, 0.293, 0.936, respectively) (Table 2, Fig. 1). On the other hand, there were significant negative correlations between serum irisin level and HbA1c and GAD-7 (r = − 0.377, − 0.435, respectively). Furthermore, significant positive correlations were found between serum testosterone, ArIIEF-5 and DM duration (r = 0.453 and 0.370, respectively) (Table 2). Meanwhile, there were statistically significant negative correlations between serum testosterone and HbA1c, TG, albumin/creatinine ratio, and GAD-7 (r = 0.408, − 0.269 and − 0.462, respectively) (Table 2). ROC analysis was conducted to predict the occurrence of ED in T2DM patients. The serum irisin biomarker had a cutoff value of < 3.755 ng/ml, with a sensitivity of 84.4% and a specificity of 75%, resulting in an AUC of 0.822 (Fig. 2). On the other hand, serum testosterone had a cutoff value of < 2.53 ng/ml, with a sensitivity of 81.3% and a specificity of 83.3%, resulting in an AUC of 0.834 (Fig. 2). Multivariate regression analysis to predict ED in T2DM patients found that GAD-7 score was the only most significant predictor for ED (ꞵ = − 1.176, standard error = 0.062, p < 0.001). In contrast, TGs and cholesterol and PHQ-9 were not significant predictors for ED in those patients (β = 0.034, standard error = 0.584, p = 0.561; β = 0.026, standard error = 0.493, p = 0.624; ꞵ = − 0.09, standard error = -– 1.28, p = 0.206, respectively).

**Table 2:** Correlations between serum irisin, testosterone, irisin-to-testosterone ratio, ArIIEF-5 scores, and different parameters among diabetic patients (n = 56)

	Serum Irisin		Serum testosterone		Irisin/T ratio		ArIIEF-5 score	
	Correlation coefficient	P value	Correlation coefficient	P value	Correlation coefficient	P value	Correlation coefficient	P value
ArIIEF-5 score	0.413	0.002	0.453	< 0.001	− 0.027	0.842	1.000	
DM duration(years)	0.293	0.028	0.370	0.005	0.091	0.504	− 0.163	0.229
BMI (kg/m2)	− 0.169	0.213	− 0.122	0.371	− 0.152	0.263	− 0.274	0.041
Hb A1c (%)	− 0.377	0.004	− 0.408	0.002	− 0.006	0.968	− 0.553	< 0.001
Triglycerides(mg/dl)	− 0.255	0.058	− 0.269	0.045	− 0.080	0.556	− 0.434	0.001
Cholesterol(mg/dl)	− 0.095	0.488	− 0.142	0.296	0.181	0.182	− 0.357	0.007
Serum Creatinine (mg/dl)	− 0.194	0.153	− 0.229	0.089	− 0.031	0.820	− 0.279	0.037
ACR	− 0.302	0.024	− 0.320	0.016	− 0.008	0.956	− 0.450	0.001
GAD-7	− 0.435	0.001	− 0.462	< 0.001	− 0.025	0.855	− 0.902	< 0.001
PHQ-9	− 0.224	0.096	− 0.229	0.090	0.045	0.739	− 0.650	< 0.001

*ACR* albumin to creatinine ratio, *BMI* body mass index, *GAD-7* generalized anxiety disorder-7, *HbA1c* glycosylated hemoglobin, *ArIIEF-5* Arabic version of the international index of erectile function-5, *PHQ-9* patient health questionnaire-9

## Discussion

The present study revealed that T2DM patients with ED exhibited significantly lower circulating serum irisin levels. A previous study had presented conflicting findings regarding the alterations in circulating serum irisin levels among individuals with T2DM, gestational diabetes mellitus, and type 1 diabetes mellitus [14].

Previous studies had indicated that diabetic patients had lower irisin levels in comparison to controls [15, 16]. In our study, the average age of the diabetic patients with ED was 49.75 years. This finding strongly supports the notion that age plays a significant role in the occurrence of ED [17]. Remarkably, patients with a short duration of T2DM might be affected, as was evident from previous studies that included males < 45 years of age [18, 19]. Moreover, the current study showed that the presence of DM was associated with more severe ED as well as lower testosterone levels. Nevertheless, an earlier study indicated no differences between T2DM patients with or without metabolic syndrome regarding ED [19, 20]. Furthermore, the present data suggest that worse glycemic control is associated with ED. Indeed, higher HbA1c levels had been correlated with ED in a previous report [21]. Furthermore, poor glycemic control had been associated with worse clinical outcomes and the emergence of many diabetic complications [22]. Through distinct pathophysiologic mechanisms, micro-angiopathy and macro-angiopathy might emerge with subsequent occurrence of ED in diabetic patients alongside autonomic neuropathy [23]. Moreover, our results revealed that age, duration of DM and triglyceride levels significantly associated with ED, as well as low irisin level, which matched a previous study [24]. Additionally, DM exerted qualitative, quantitative, and kinetic abnormalities on lipoproteins and increased TG content as well as glycation of apolipoproteins [25]. Circulating irisin was significantly reduced in T2DM patients compared with healthy controls [15], which could be seen in line with our results. Our study demonstrated that reduced serum irisin levels were independently associated with elevated HbA1c. This finding might be explained as follows.

Firstly, a negative association between serum irisin and hyperglycemia and triglyceridemia as well as visceral adiposity in T2DM patient had been demonstrated by Kurdiova et al. [26]. In similar trend, a previous study showed that serum irisin directly induced glucose and fatty acid uptake in human muscles via the AMPK pathway [6]. Moreover, the over-expression of this myokine was sufficient to promote energy expenditure and alleviate insulin resistance in a diabetic animal model [7]. Further, a previous study reported that irisin played a novel role in sustaining endothelial homoeostasis through the promotion of human umbilical vein endothelial cell proliferation via the extracellular signal-regulated ERK signaling pathway [27]. Consistently, a modern study that was conducted on mice had revealed that serum irisin had protective effects on endothelia in type 2 diabetes, partially through reducing oxidative/nitrative stress and inhibiting activation of PKC-β/ NADPH oxidase and NF-κB/iNOS pathways [28]. Thus, in view of the aforementioned findings it could be postulated that irisin might be effective in the treatment of diabetic vascular complications [28]. Regarding the current results, irisin levels were found to be significantly lower among T2DM individuals with ED when compared to those with normal erectile function. This finding suggests that irisin can potentially have a protective impact on endothelial function that could be seen in alignment with the aforementioned studies [8, 27, 28]. Furthermore, Zhang et al. [29] suggested that irisin might be a possible marker of macrovascular disease in T2DM patients. A modern study had demonstrated that irisin played an important role in maintaining endothelial cell function, and low levels of irisin led to endothelial dysfunction and an increased incidence of atherosclerosis [30]. Furthermore, a previous study had indicated that irisin could mitigate endothelial damage by suppressing inflammation and oxidative stress [8].

Remarkably, the current study had demonstrated a positive correlation between serum irisin and serum testosterone level that could be seen in line with two previous studies [31, 32]. However, there was no significant association between serum irisin level and duration of DM in the current study. This lack of association may be attributed to the limited size of our study sample. Similarly, another study also failed to find a significant relationship between diabetes duration and ED [33]. Quite the reverse, previous studies demonstrated significant negative correlations [34, 35]. In the same context, a recent study showed that the risk of ED increased with DM duration of more than 10 years as well as being uncontrolled [36]. Furthermore, the present study revealed a negative correlation between serum irisin and ArIIEF-5, PHQ-9, and GAD-7 scores. This observation aligns with earlier studies conducted by Mallis et al. [37] and Yang et al. [38]. Consistently, this could be seen in agreement with Ragab et al. [39] who revealed that serum nesfatin 1 in ED diabetic patients significantly correlated with GAD-7. Admittedly, the current study had some shortcomings, including a small sample size and a brief duration of monitoring, which may be considered substantial limitations.

Remarkably, the current study had demonstrated a positive correlation between serum irisin and serum testosterone level that could be seen in line with two previous studies [31, 32]. However, there was no significant association between serum irisin level and duration of DM in the current study. This lack of association may be attributed to the limited size of our study sample. Similarly, another study also failed to find a significant relationship between diabetes duration and ED [33]. Quite the reverse, previous studies demonstrated significant negative correlations [34, 35]. In the same context, a recent study showed that the risk of ED increased with DM duration of more than 10 years as well as being uncontrolled [36]. Furthermore, the present study revealed a negative correlation between serum irisin and ArIIEF-5, PHQ-9, and GAD-7 scores. This observation aligns with earlier studies conducted by Mallis et al. [37] and Yang et al. [38]. Consistently, this could be seen in agreement with Ragab et al. [39] who revealed that serum nesfatin 1 in ED diabetic patients significantly correlated with GAD-7. Admittedly, the current study had some shortcomings, including a small sample size and a brief duration of monitoring, which may be considered substantial limitations.

## Conclusion

The current study had demonstrated that irisin positively correlated with the ArIIEF-5 and serum testosterone but negatively correlated with HbA1c in T2DM men. Nevertheless, further validation of these findings is necessary through cohort studies.

## Supplementary Information

Supplementary Material 1.

## Data Availability

The data that support the fndings of this study are available from the cor responding author upon reasonable request.
