# Supplementary material for: Evaluation of serum irisin level and severity of erectile dysfunction in diabetic males: a cross sectional prospective study
Source: Diabetol Metab Syndr. 2024 Sep 19;16:233. doi: 10.1186/s13098-024-01452-3 (PMC11412007; doi:10.1186/s13098-024-01452-3)
Supplement: Supplementary file 1 — Supplementary Material 1. [file 13098_2024_1452_MOESM1_ESM.docx]

|  | | Group I  [DM with ED]  n =32 | | Group II  [DM without ED] n=24 | | Group III  [Controls]  n=34 | | P value |
| --- | --- | --- | --- | --- | --- | --- | --- | --- |
|  |  | Count | % | Count | % | Count | % |  |
| Special habits | Ex-smoker | 20 | 62.5% | 7 | 29.2% | 6 | 17.6% | 0.001 |
|  | Non-smoker | 12 | 37.5% | 17 | 70.8% | 28 | 82.4% |  |
| DM complications | Retinopathy | 2 | 6.3% | 1 | 4.2% | 0 | 0.0% | 0.136 |
|  | Neuropathy | 5 | 15.6% | 0 | 0.0% | 0 | 0.0% |  |
|  | Nephropathy | 3 | 9.4% | 1 | 4.2% | 0 | 0.0% |  |
|  | No | 22 | 68.7% | 22 | 91.6% | 34 | 100.0% |  |
| Co-morbidities | Peripheral vascular disease | 3 | 9.4% | 0 | 0.0% | 0 | 0.0% | 0.331 |
|  | Hypertension | 3 | 9.4% | 1 | 4.2% | 0 | 0.0% |  |
|  | No | 26 | 81.3% | 23 | 95.8% | 0 | 0.0% |  |

Table (1) shows medical history of all participants

Abbreviations: DM- diabetes mellitus; ED- erectile dysfunction

Table (2) shows the scores of generalized anxiety disorder-7 (GAD-7), patient health questionnaire-9 (PHQ-9) and Arabic version of the international index of erectile function-5 (IIEF-5) among all participants

|  | | Group I  [DM with ED]  n =32 | | Group II  [DM without ED] n=24 | | Group III  [Controls]  n=34 | | P value |
| --- | --- | --- | --- | --- | --- | --- | --- | --- |
|  |  | Count | % | Count | % | Count | % |  |
| GAD-7 result | Minimal anxiety | 0 | 0.0% | 19 | 79.2% | 34 | 100.0% | < 0.001 |
|  | Mild anxiety | 8 | 25.0% | 5 | 20.8% | 0 | 0.0% |  |
|  | Moderate anxiety | 21 | 65.6% | 0 | 0.0% | 0 | 0.0% |  |
|  | Severe anxiety | 3 | 9.4% | 0 | 0.0% | 0 | 0.0% |  |
| PHQ-9 result | Mild depression | 15 | 46.9% | 3 | 12.5% | 1 | 2.9% | < 0.001 |
|  | Moderate depression | 6 | 18.8% | 0 | 0.0% | 0 | 0.0% |  |
|  | Moderately severe | 3 | 9.4% | 0 | 0.0% | 0 | 0.0% |  |
|  | No depression | 8 | 25.0% | 21 | 87.5% | 33 | 97.1% |  |
| ArIIEF-5 score | Normal | 0 | 0.0% | 24 | 100.0% | 34 | 100.0% | < 0.001 |
|  | Mild ED | 5 | 15.6% | 0 | 0.0% | 0 | 0.0% |  |
|  | Mild to moderate ED | 8 | 25.0% | 0 | 0.0% | 0 | 0.0% |  |
|  | Moderate ED | 16 | 50.0% | 0 | 0.0% | 0 | 0.0% |  |
|  | Severe ED | 3 | 9.4% | 0 | 0.0% | 0 | 0.0% |  |

Abbreviations: ArIIEF-5- Arabic version of the international index of erectile function-5;DM- diabetes mellitus; ED- erectile dysfunction; GAD-7-generalized anxiety disorder-7; PHQ-9- patient health questionnaire-9
